# Synthesis, crystal structure and Hirshfeld surface analysis of [Cu(NO_3_)_2_{8-methyl­phenanthridin-6(5*H*)-one}_4_]

**DOI:** 10.1107/S2056989025009892

**Published:** 2025-11-11

**Authors:** Atash V. Gurbanov, Tuncer Hökelek, Menberu Mengesha Woldemariam

**Affiliations:** a Excellence Center, Baku State University, Z. Khalilov Str. 33, AZ1148, Baku, Azerbaijan; b Hacettepe University, Department of Physics, 06800 Beytepe-Ankara, Türkiye; c Department of Physics, Jimma University, Jimma, Ethiopia; Universidade Federal do ABC, Brazil

**Keywords:** copper complexes, N-containing compounds, crystal structure, non-covalent inter­actions

## Abstract

The asymmetric unit of the title compound, tetra­kis­[8-methyl­phenanthridin-6(5*H*)-one-κ*O*]bis­(nitrato-κ*O*)copper(II), [Cu(NO_3_)_2_(C_14_H_11_NO)_4_], contains one Cu^II^ cation located on a centre of symmetry, two 8-methyl­phenanthridin-6(5*H*)-one (MPHNT) ligands and one nitrate anion, where the Cu^II^ atom is in a slightly distorted octa­hedral environment. Intra­molecular N—H⋯O hydrogen bonds are observed. In the crystal, C—H⋯O hydrogen bonds link the mol­ecules, enclosing S(6), S(9) and *R*^4^_4_(18) ring motifs. In addition π–π inter­actions with centroid-to-centroid distances of 3.808 (2) Å and also a series of C—H⋯π(ring) inter­actions help to consolidate the packing in a three-dimensional architecture within the crystal. A Hirshfeld surface analysis revealed that the most important contributions for the crystal packing are from H⋯H (41.1%), H⋯C/C⋯H (29.9%), H⋯O/O⋯H (14.8%) and C⋯C (10.0%) inter­actions.

## Abstract 2

The asymmetric unit of the title compound, C_56_H_44_CuN_6_O_10_, contains one half of the complex mol­ecule. In the crystal, C—H⋯O hydrogen bonds link the mol­ecules, enclosing *R*^4^_4_(18) ring motifs. In addition π–π inter­actions with centroid-to-centroid distances of 3.808 (2) Å and also a series of C—H⋯π(ring) inter­actions help to consolidate the packing in a three-dimensional architecture within the crystal.

## 1. Chemical context

N-containing organic compounds are a widely used and versatile class of ligands in coordination chemistry due to the nitro­gen atom’s strong σ-donor characteristics, which stabilize various metal oxidation states (Gurbanov *et al.*, 2023 ▸; Mahmudov *et al.*, 2021 ▸, 2023 ▸; Kretschmer, 2020 ▸; Peris, 2018 ▸). These ligands are employed in diverse applications, including mol­ecular recognition, homogenous catalysis, crystal engineering, material science, organic synthesis and medicinal chemistry (Gadzhieva *et al.*, 2005 ▸; Maharramov *et al.*, 2011 ▸; Gurbanov *et al.*, 2022 ▸). Alteration of the metal centre as well as substituents at the N-ligands dictate the sensing and analytical properties, catalytic activity, and supra­molecular arrangements of the corresponding metal complexes (Aliyeva *et al.*, 2024 ▸; Gurbanov *et al.*, 2018 ▸; Huseynov *et al.*, 2018 ▸). In particular, the coordination chemistry of copper with N-ligands is extremely rich due to its oxidation states Cu^0^, Cu^I^, Cu^II^ and Cu^III^ allowing it to act through one- or two-electron processes in organic transformations (Allen *et al.*, 2013 ▸). We have synthesized a new copper(II) complex with an 8-methyl­phenanthridin-6(5*H*)-one ligand, which is consolidated through intra- and inter­molecular non-covalent inter­actions. Herein, we report its synthesis and mol­ecular and crystal structures together with the results of a Hirshfeld surface analysis.
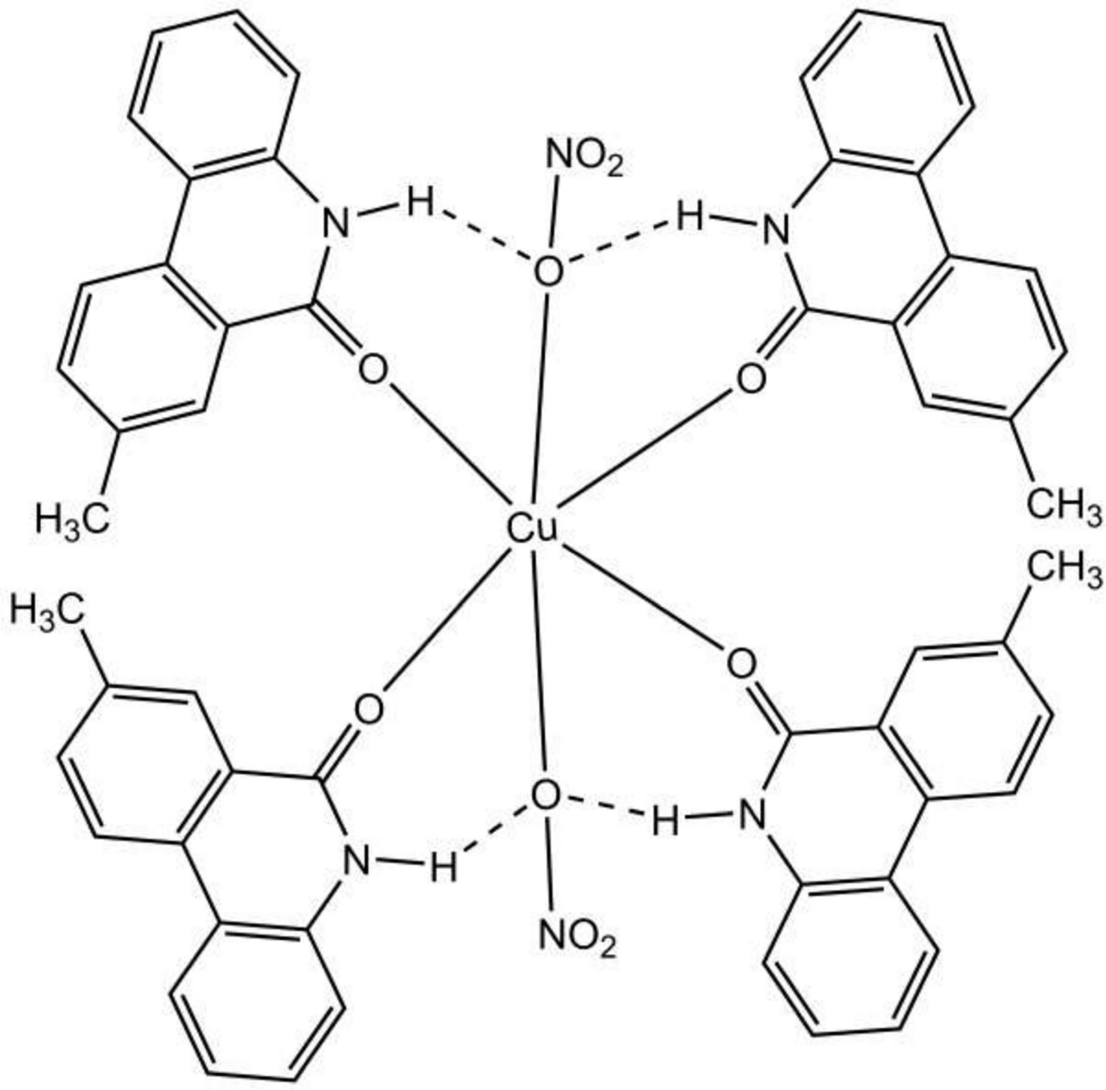


## 2. Structural commentary

**Figure 1:**
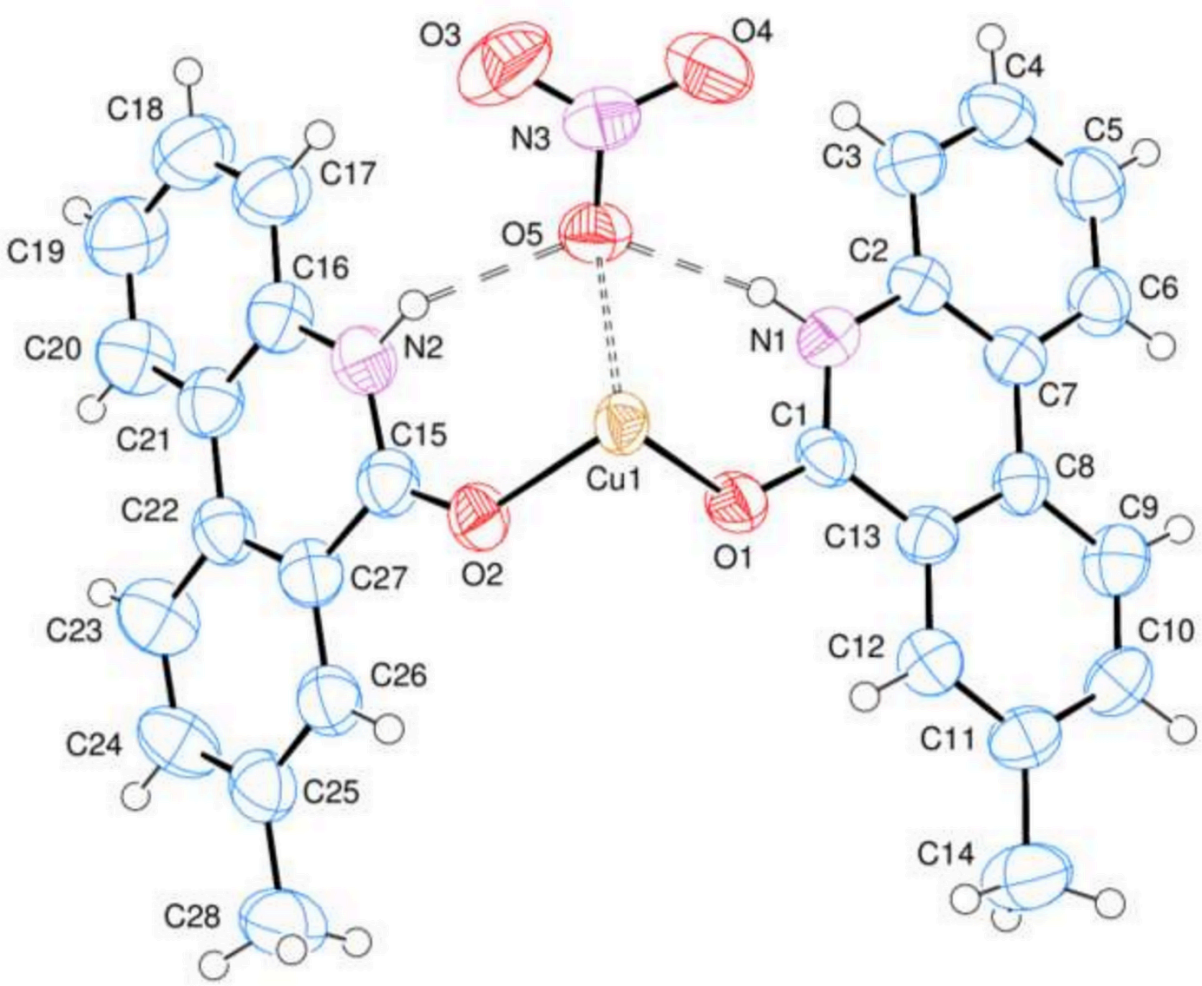
The asymmetric unit of the title compound with the atom-numbering scheme and 50% probability ellipsoids. Intra­molecular N—H⋯O hydrogen bonds are shown as dashed lines.

The asymmetric unit of the title compound, C_56_H_44_CuN_6_O_10_, (I) contains one Cu^II^ cation located on a crystallographic inversion centre, two 8-methyl­phenanthridin-6(5*H*)-one (MPHNT) and one nitrate anion (Fig. 1 ▸). The Cu^II^ atom is in a slightly distorted octa­hedral environment and is coordinated by four symmetry-related MPHNT O atoms (O1, O2 and O1′, O2′) in the basal plane at distances of 1.953 (2) and 1.942 (2) Å in a square-planar arrangement and by two symmetry-related O atoms (O5 and O5′>) at distances of 2.520 (3) Å in the axial positions (Table 1 ▸) [symmetry code: (′) 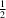 − *x*, 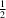 − *y*, −*z* + 1]. The phenanthridin ring systems [(*A* (N1/C1–C13) and (*B* (N2/C15–C27)] are essentially planar with r.m.s. deviations of 0.03 (4) and 0.03 (6) Å, respectively (Fig. 1 ▸). Atoms O1, O2, C14 and C28 are −0.006 (3), 0.182 (3), 0.046 (6) and 0.100 (6) Å away from the best least-squares planes of the corresponding ring systems. The ring systems are oriented at a dihedral angle of *A*/*B* = 68.97 (6)°. Intra­molecular N—H⋯O hydrogen bonds (Table 2 ▸) occur between N atoms of MPHNT and O atoms of nitrate anions (Fig. 1 ▸).

## 3. Supra­molecular features

**Figure 2:**
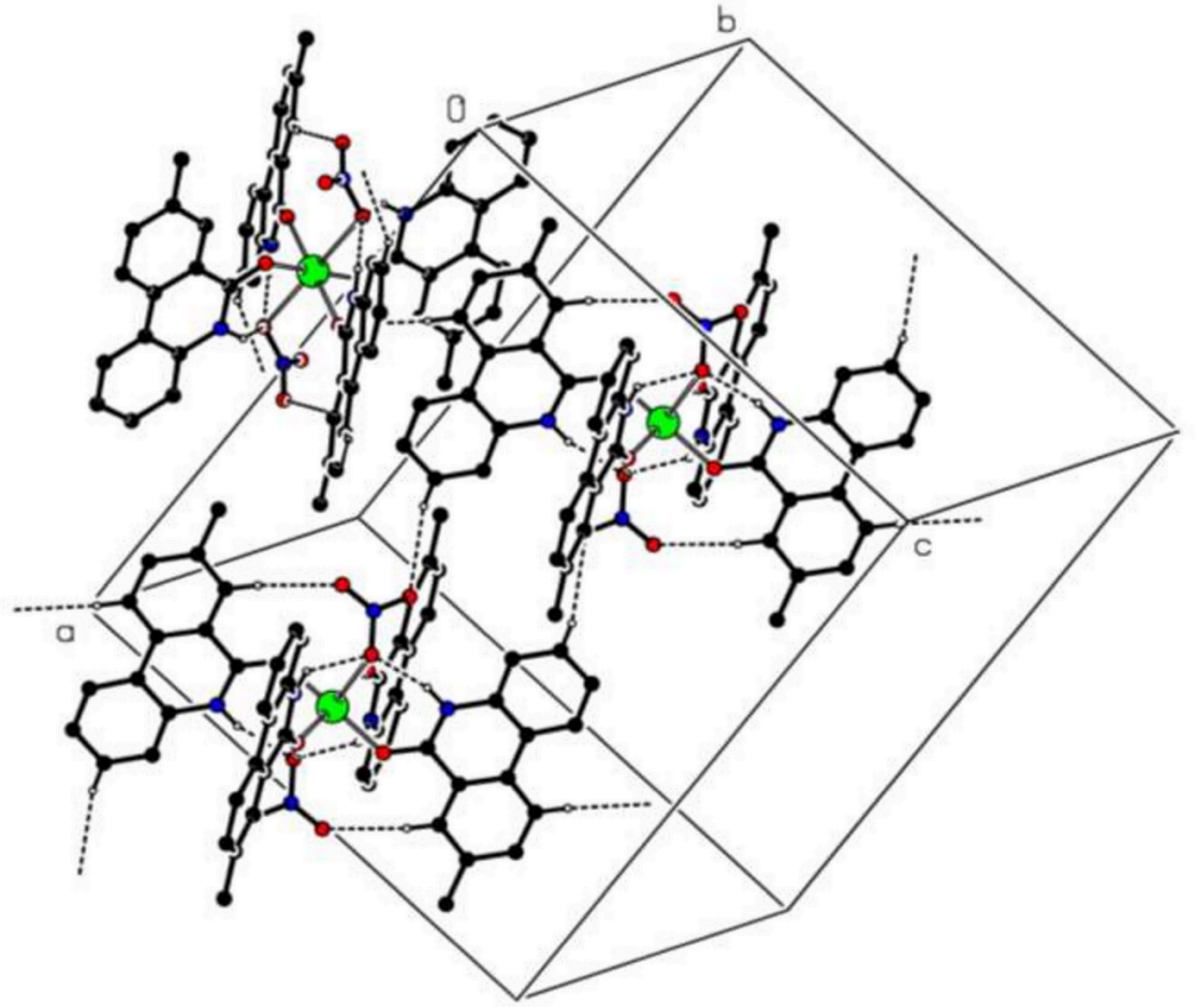
The partial packing diagram of the title compound. Intra­molecular N—H⋯O and inter­molecular C—H⋯O hydrogen bonds are shown as dashed lines. H atoms not involved in these inter­actions been omitted for clarity.

In the crystal, C—H⋯O hydrogen bonds (Table 2 ▸) link the mol­ecules, enclosing *S*(6), *S*(9) and 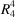
(18) ring motifs (Etter *et al.*, 1990 ▸) (Fig. 2 ▸). In addition, π–π inter­actions between (N2/C15/C16/C21/C22/C27) and (C22–C27) rings with centroid–to–centroid distances of 3.808 (2) Å [where α = 0.8 (2)° and slippage = 1.592 Å] and a series of the C—H⋯π(ring) inter­actions (Table 2 ▸) help to consolidate the packing in a three-dimensional architecture within the crystal.

## 4. Hirshfeld surface analysis

**Figure 3:**
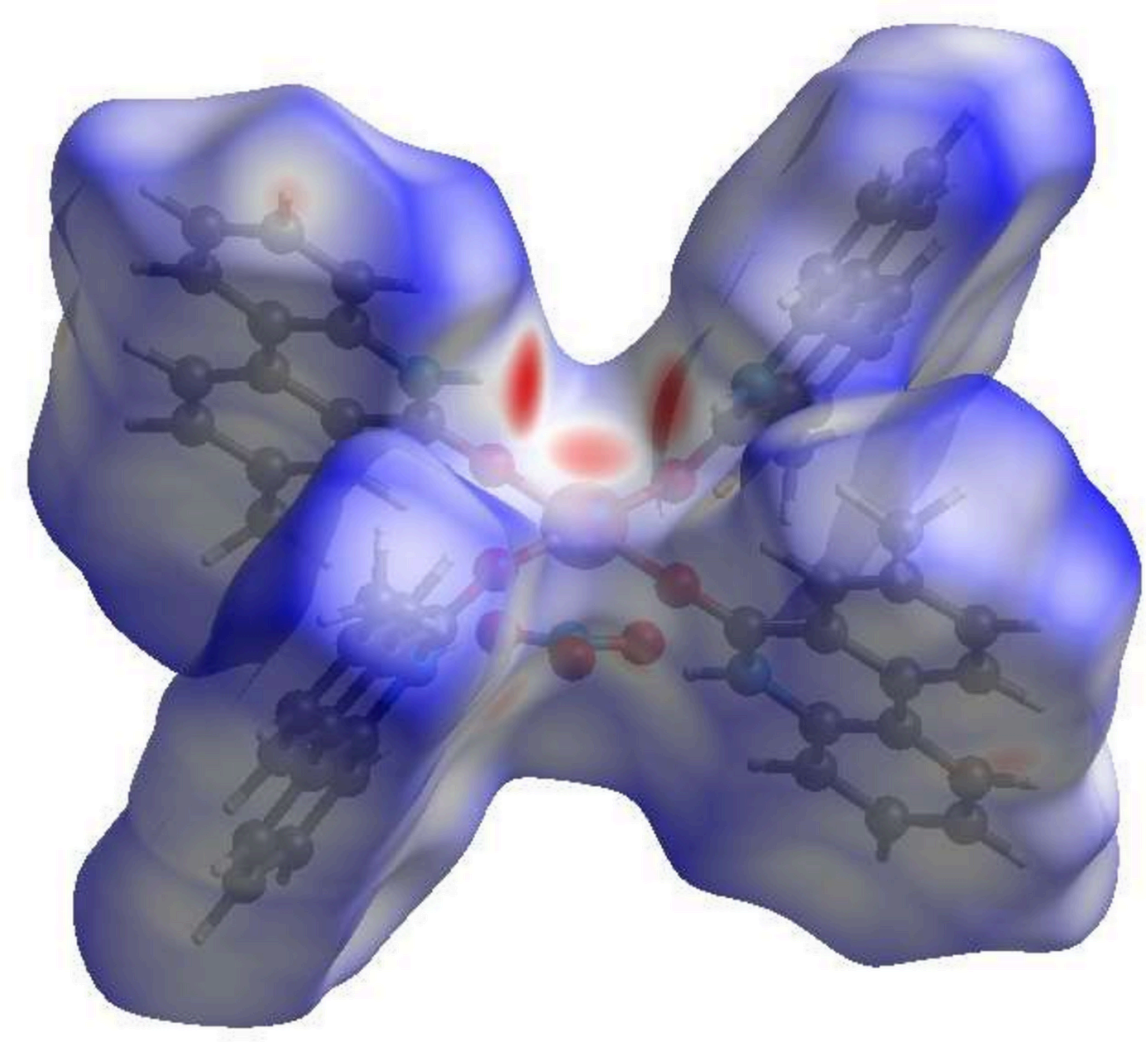
View of the three-dimensional Hirshfeld surface plotted over *d*_norm_.

**Figure 4:**
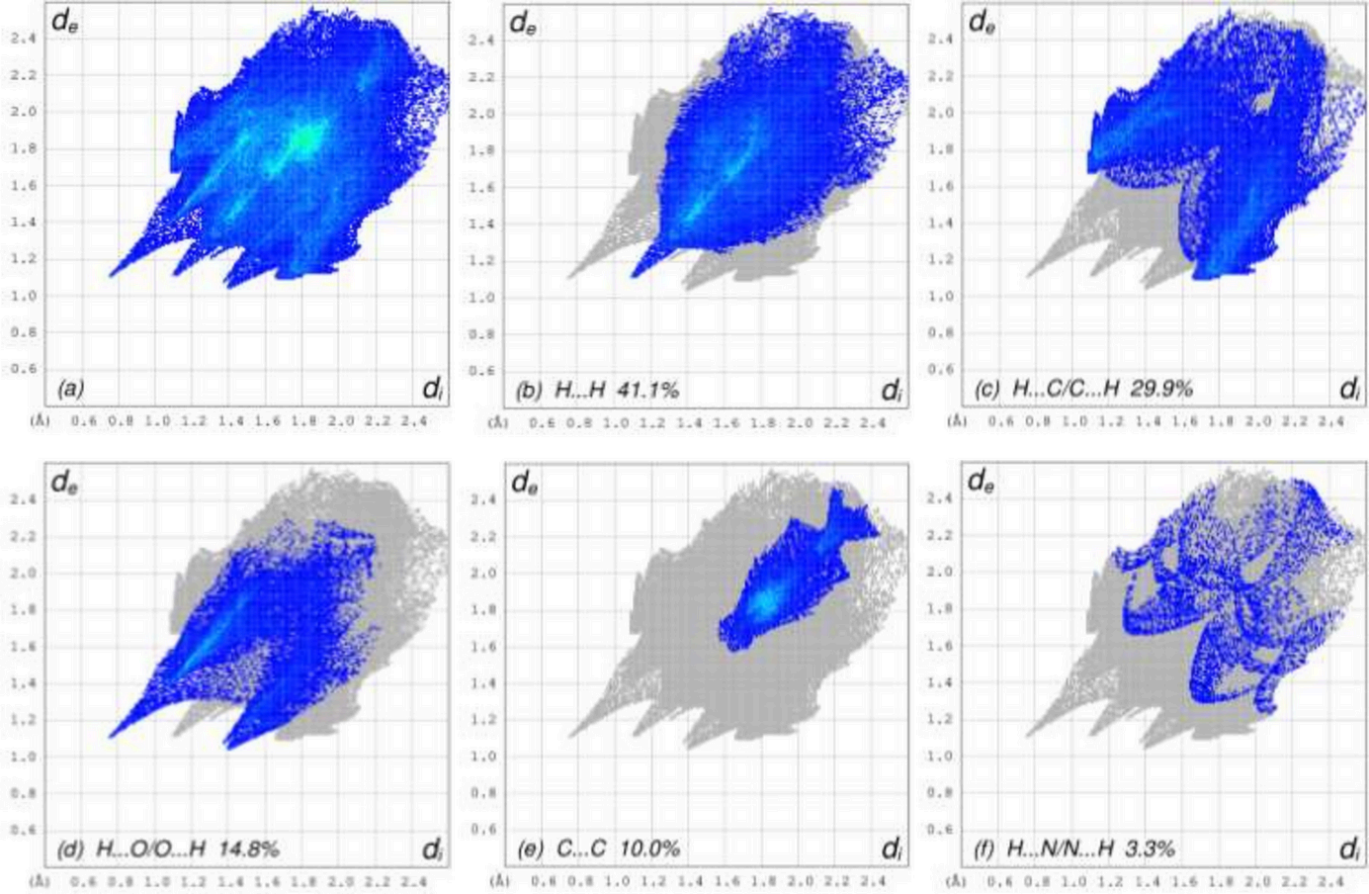
The full two-dimensional fingerprint plots, showing (*a*) all inter­actions, and delineated into (*b*) H⋯H, (*c*) H⋯C/C⋯H, (*d*) H⋯O/O⋯H, (*e*) C⋯C, (*f*) H⋯N/N⋯H, (*g*) C⋯N/N⋯C, (*h*) O⋯O, (*i*) O⋯Cu/Cu⋯O and (*j*) N⋯O/O⋯N inter­actions. The *d*_i_ and *d*_e_ values are the closest inter­nal and external distances (in Å) from given points on the Hirshfeld surface.

To visualize the inter­molecular inter­actions in the title compound, a Hirshfeld surface (HS) analysis was carried out using *Crystal Explorer 17.5* (Spackman *et al.*, 2021 ▸). In the HS plotted over *d*_norm_ (Fig. 3 ▸), the contact distances equal, shorter and longer than the sum of van der Waals radii are shown by the white, red and blue colours, respectively. According to the two-dimensional fingerprint plots, H⋯H, H⋯C/C⋯H, H⋯O/O⋯H and C⋯C contacts make the most important contributions to the HS (Fig. 4 ▸).

## 5. Synthesis and crystallization

832 mg (4.0 mmol) of 8-methyl­phenanthridin-6(5H)-one were dissolved in 100 mL of ethanol and 233 mg (1.0 mmol) of Cu(NO_3_)_2_·2.5H_2_O were added with stirring. The mixture was stirred for 5 min and left standing for slow solvent evaporation. Brown crystals started to form in the reaction mixture after 2 d at room temperature. After 3 d they were filtered off and dried in air. Yield: 55% (based on Cu). Analysis calculated for C_56_H_44_CuN_6_O_10_ (*M* = 1024.55): C, 65.65; H, 4.33; N, 8.20. Found: C 65.62; H, 4.30; N, 8.17%. IR, cm^−1^: 3212 ν(N—H) and 1648 ν(C=O).

## 6. Refinement

Crystal data, data collection and structure refinement details are summarized in Table 3 ▸. The N- and C-bound H-atom positions were calculated geometrically at distances of 0.90 (for NH), 0.93 (for aromatic CH) and 0.96 Å (for CH_3_) and refined using a riding model by applying the constraint of *U*_iso_(H) = *k* × *U*_eq_(C,N), where *k* = 1.5 for methyl H atoms and k = 1.2 for the other H atoms.

## Supplementary Material

Crystal structure: contains datablock(s) I, global. DOI: 10.1107/S2056989025009892/ee2022sup1.cif

Structure factors: contains datablock(s) I. DOI: 10.1107/S2056989025009892/ee2022Isup2.hkl

CCDC reference: 2501014

Additional supporting information:  crystallographic information; 3D view; checkCIF report

## Figures and Tables

**Table 1:** Selected geometric parameters (Å, °)

Cu1—O2	1.942 (2)	Cu1—O5	2.520 (3)
Cu1—O1	1.953 (2)		
			
O2^ii^—Cu1—O1	90.74 (11)	O1—Cu1—O5	90.79 (11)
O2—Cu1—O1	89.26 (11)	O2—Cu1—O5	94.21 (11)

Symmetry codes: (i) 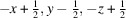; (ii) 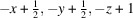.

**Table 2:** Hydrogen-bond geometry (Å, °) *Cg*1, *Cg*3, *Cg*4 and *Cg*5 are the centroids of the (N1/C1/C2/C7/C8/C13), (C2–C7), (C8–C13) and (C16–C21) rings, respectively.

*D*—H⋯*A*	*D*—H	H⋯*A*	*D*⋯*A*	*D*—H⋯*A*
N1—H1*N*⋯O5	0.90	1.97	2.852 (4)	165
N2—H2*N*⋯O5	0.90	2.07	2.806 (4)	139
C4—H4*A*⋯O4^iii^	0.93	2.60	3.433 (6)	150
C9—H9*A*⋯O1^i^	0.93	2.60	3.514 (5)	169
C12—H12*A*⋯O3^ii^	0.93	2.57	3.455 (6)	158
C14—H14*C*⋯*Cg*5^vi^	0.96	2.92	3.768 (6)	147
C20—H20*A*⋯*Cg*3^vii^	0.93	2.86	3.644 (5)	143
C23—H23*A*⋯*Cg*1^vii^	0.93	2.89	3.726 (5)	151
C24—H24*A*⋯*Cg*4^vii^	0.93	2.77	3.557 (5)	143
C28—H28*C*⋯*Cg*3^viii^	0.96	2.88	3.716 (6)	146

Symmetry codes: (i) 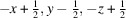; (ii) 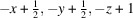; (iii) 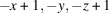; (vi) 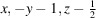; (vii) 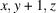; (viii) 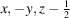.

**Table 3:** Experimental details

Crystal data	
Chemical formula	[Cu(NO_3_)_2_(C_14_H_11_NO)_4_]
*M*_r_	1024.51
Crystal system, space group	Monoclinic, *C*2/*c*
Temperature (K)	296
*a*, *b*, *c* (Å)	22.279 (2), 11.9230 (11), 18.1267 (16)
β (°)	102.444 (4)
*V* (Å^3^)	4701.9 (8)
*Z*	4
Radiation type	Mo *K*α
μ (mm^−1^)	0.54
Crystal size (mm)	0.26 × 0.21 × 0.11

Data collection	
Diffractometer	Bruker APEXII CCD
Absorption correction	Multi-scan (*SADABS*; Krause *et al.*, 2015 ▸)
*T*_min_, *T*_max_	0.861, 0.934
No. of measured, independent and observed [*I* > 2σ(*I*)] reflections	31022, 4623, 2755
*R*_int_	0.087
(sin θ/λ)_max_ (Å^−1^)	0.617

Refinement	
*R*[*F*^2^ > 2σ(*F*^2^)], *wR*(*F*^2^), *S*	0.060, 0.184, 1.02
No. of reflections	4623
No. of parameters	333
H-atom treatment	H-atom parameters constrained
Δρ_max_, Δρ_min_ (e Å^−3^)	0.64, −0.28

Computer programs: *APEX4* and *SAINT* (Bruker, 2016 ▸), *SHELXT2019/1* (Sheldrick, 2015*a* ▸), *SHELXL2019/1* (Sheldrick, 2015*b* ▸), *ORTEP-3 for Windows* and *WinGX* publication routines (Farrugia, 2012 ▸) and *PLATON* (Spek, 2020 ▸).

## supplementary crystallographic information

### Tetrakis[8-methylphenanthridin-6(5H)-one-κO]bis(nitrato-κO)copper(II) Crystal data

**Table:**

[Cu(NO_3_)_2_(C_14_H_11_NO)_4_]	*F*(000) = 2124
*M**_r_* = 1024.51	*D*_x_ = 1.447 Mg m^−^^3^
Monoclinic, *C*2/*c*	Mo *K*α radiation, λ = 0.71073 Å
*a* = 22.279 (2) Å	Cell parameters from 3386 reflections
*b* = 11.9230 (11) Å	θ = 3.2–20.3°
*c* = 18.1267 (16) Å	µ = 0.54 mm^−^^1^
β = 102.444 (4)°	*T* = 296 K
*V* = 4701.9 (8) Å^3^	Prism, brown
*Z* = 4	0.26 × 0.21 × 0.11 mm

### Tetrakis[8-methylphenanthridin-6(5H)-one-κO]bis(nitrato-κO)copper(II) Data collection

**Table:**

Bruker APEXII CCD diffractometer	2755 reflections with *I* > 2σ(*I*)
φ and ω scans	*R*_int_ = 0.087
Absorption correction: multi-scan (SADABS; Krause *et al.*, 2015)	θ_max_ = 26.0°, θ_min_ = 3.2°
*T*_min_ = 0.861, *T*_max_ = 0.934	*h* = −27→27
31022 measured reflections	*k* = −14→14
4623 independent reflections	*l* = −22→22

### Tetrakis[8-methylphenanthridin-6(5H)-one-κO]bis(nitrato-κO)copper(II) Refinement

**Table:**

Refinement on *F*^2^	Primary atom site location: structure-invariant direct methods
Least-squares matrix: full	Secondary atom site location: difference Fourier map
*R*[*F*^2^ > 2σ(*F*^2^)] = 0.060	Hydrogen site location: mixed
*wR*(*F*^2^) = 0.184	H-atom parameters constrained
*S* = 1.02	*w* = 1/[σ^2^(*F*_o_^2^) + (0.0967*P*)^2^ + 2.2853*P*] where *P* = (*F*_o_^2^ + 2*F*_c_^2^)/3
4623 reflections	(Δ/σ)_max_ < 0.001
333 parameters	Δρ_max_ = 0.64 e Å^−^^3^
0 restraints	Δρ_min_ = −0.28 e Å^−^^3^

### Tetrakis[8-methylphenanthridin-6(5H)-one-κO]bis(nitrato-κO)copper(II) Special details

**Table:**

Geometry. All esds (except the esd in the dihedral angle between two l.s. planes) are estimated using the full covariance matrix. The cell esds are taken into account individually in the estimation of esds in distances, angles and torsion angles; correlations between esds in cell parameters are only used when they are defined by crystal symmetry. An approximate (isotropic) treatment of cell esds is used for estimating esds involving l.s. planes.

### Tetrakis[8-methylphenanthridin-6(5H)-one-κO]bis(nitrato-κO)copper(II) Fractional atomic coordinates and isotropic or equivalent isotropic displacement parameters (Å^2^)

**Table:**

	*x*	*y*	*z*	*U*_iso_*/*U*_eq_	
Cu1	0.250000	0.250000	0.500000	0.0399 (2)	
O1	0.23229 (12)	0.2038 (2)	0.39416 (14)	0.0487 (7)	
O2	0.23037 (12)	0.4036 (2)	0.46793 (15)	0.0490 (7)	
O3	0.42140 (18)	0.2859 (4)	0.6104 (2)	0.0918 (12)	
O4	0.41796 (16)	0.1304 (3)	0.5488 (2)	0.0909 (12)	
O5	0.36260 (13)	0.2693 (2)	0.49965 (16)	0.0557 (8)	
N1	0.31896 (15)	0.1148 (3)	0.38002 (19)	0.0510 (9)	
H1N	0.339116	0.157220	0.418564	0.061*	
N2	0.32132 (17)	0.4913 (3)	0.5025 (2)	0.0593 (10)	
H2N	0.335582	0.427329	0.526291	0.071*	
N3	0.40159 (17)	0.2283 (3)	0.5554 (2)	0.0583 (10)	
C1	0.25888 (18)	0.1347 (3)	0.3591 (2)	0.0440 (9)	
C2	0.35192 (18)	0.0411 (3)	0.3432 (2)	0.0470 (10)	
C3	0.4148 (2)	0.0288 (4)	0.3704 (3)	0.0627 (12)	
H3A	0.434875	0.069264	0.412391	0.075*	
C4	0.4475 (2)	−0.0448 (4)	0.3342 (3)	0.0687 (13)	
H4A	0.489718	−0.052775	0.351440	0.082*	
C5	0.4174 (2)	−0.1058 (4)	0.2727 (3)	0.0694 (13)	
H5A	0.439192	−0.155812	0.249106	0.083*	
C6	0.3553 (2)	−0.0929 (4)	0.2464 (3)	0.0593 (12)	
H6A	0.335682	−0.134016	0.204511	0.071*	
C7	0.32065 (18)	−0.0194 (3)	0.2808 (2)	0.0475 (10)	
C8	0.25515 (18)	−0.0035 (3)	0.2560 (2)	0.0437 (9)	
C9	0.2184 (2)	−0.0650 (4)	0.1974 (2)	0.0587 (12)	
H9A	0.236506	−0.119799	0.172896	0.070*	
C10	0.1568 (2)	−0.0466 (4)	0.1753 (2)	0.0590 (11)	
H10A	0.134131	−0.087837	0.135259	0.071*	
C11	0.1266 (2)	0.0326 (4)	0.2113 (2)	0.0556 (11)	
C12	0.16139 (19)	0.0903 (3)	0.2707 (2)	0.0499 (10)	
H12A	0.142317	0.141936	0.296581	0.060*	
C13	0.22473 (18)	0.0737 (3)	0.2935 (2)	0.0443 (9)	
C14	0.0588 (2)	0.0528 (5)	0.1853 (3)	0.0811 (15)	
H14A	0.044342	0.095921	0.222726	0.122*	
H14B	0.037661	−0.017822	0.178320	0.122*	
H14C	0.051105	0.093187	0.138434	0.122*	
C15	0.2617 (2)	0.4901 (3)	0.4683 (2)	0.0508 (10)	
C16	0.3621 (2)	0.5793 (4)	0.4979 (2)	0.0570 (11)	
C17	0.4239 (2)	0.5659 (4)	0.5316 (3)	0.0736 (14)	
H17A	0.437657	0.500156	0.557358	0.088*	
C18	0.4649 (3)	0.6521 (5)	0.5264 (3)	0.0861 (16)	
H18A	0.506435	0.644243	0.548237	0.103*	
C19	0.4429 (3)	0.7502 (5)	0.4883 (3)	0.0865 (16)	
H19A	0.469955	0.808523	0.484932	0.104*	
C20	0.3808 (3)	0.7613 (4)	0.4551 (3)	0.0711 (14)	
H20A	0.367097	0.827183	0.429407	0.085*	
C21	0.3388 (2)	0.6769 (4)	0.4592 (2)	0.0592 (11)	
C22	0.2743 (2)	0.6844 (3)	0.4257 (2)	0.0551 (11)	
C23	0.2475 (3)	0.7787 (4)	0.3877 (3)	0.0724 (14)	
H23A	0.271265	0.842018	0.384730	0.087*	
C24	0.1844 (3)	0.7790 (4)	0.3534 (3)	0.0712 (14)	
H24A	0.167386	0.842725	0.327463	0.085*	
C25	0.1475 (2)	0.6888 (4)	0.3572 (3)	0.0652 (13)	
C26	0.1735 (2)	0.5943 (4)	0.3975 (2)	0.0576 (11)	
H26A	0.148801	0.532905	0.401998	0.069*	
C27	0.2356 (2)	0.5913 (3)	0.4307 (2)	0.0525 (10)	
C28	0.0813 (2)	0.6927 (5)	0.3189 (3)	0.0905 (17)	
H28A	0.068979	0.769309	0.308519	0.136*	
H28B	0.057088	0.659934	0.351196	0.136*	
H28C	0.075071	0.651528	0.272393	0.136*	

### Tetrakis[8-methylphenanthridin-6(5H)-one-κO]bis(nitrato-κO)copper(II) Atomic displacement parameters (Å^2^)

**Table:**

	*U*^11^	*U*^22^	*U*^33^	*U*^12^	*U*^13^	*U*^23^
Cu1	0.0504 (4)	0.0317 (3)	0.0358 (4)	−0.0007 (3)	0.0054 (3)	0.0007 (3)
O1	0.0540 (17)	0.0476 (14)	0.0409 (15)	0.0093 (14)	0.0027 (13)	−0.0027 (13)
O2	0.0569 (17)	0.0347 (14)	0.0542 (16)	−0.0018 (13)	0.0092 (14)	0.0065 (12)
O3	0.081 (3)	0.127 (3)	0.060 (2)	−0.011 (2)	−0.0015 (19)	−0.025 (2)
O4	0.068 (2)	0.073 (2)	0.122 (3)	0.0179 (19)	−0.002 (2)	0.013 (2)
O5	0.0504 (18)	0.0598 (18)	0.0530 (17)	0.0034 (14)	0.0028 (14)	0.0067 (13)
N1	0.048 (2)	0.053 (2)	0.049 (2)	−0.0009 (16)	0.0032 (17)	−0.0049 (16)
N2	0.064 (3)	0.0394 (19)	0.070 (2)	−0.0005 (17)	0.004 (2)	0.0080 (17)
N3	0.043 (2)	0.070 (3)	0.061 (2)	−0.0010 (19)	0.0082 (19)	0.0013 (19)
C1	0.050 (3)	0.039 (2)	0.041 (2)	0.0074 (18)	0.0065 (19)	0.0061 (17)
C2	0.046 (2)	0.048 (2)	0.048 (2)	0.0030 (19)	0.012 (2)	0.0027 (18)
C3	0.056 (3)	0.066 (3)	0.064 (3)	0.000 (2)	0.007 (2)	0.001 (2)
C4	0.051 (3)	0.072 (3)	0.085 (4)	0.009 (2)	0.018 (3)	0.008 (3)
C5	0.067 (3)	0.065 (3)	0.081 (3)	0.006 (3)	0.027 (3)	−0.004 (3)
C6	0.061 (3)	0.055 (3)	0.065 (3)	0.004 (2)	0.020 (2)	−0.009 (2)
C7	0.049 (3)	0.049 (2)	0.046 (2)	0.0028 (19)	0.012 (2)	0.0031 (18)
C8	0.052 (2)	0.040 (2)	0.040 (2)	−0.0011 (18)	0.0122 (18)	−0.0007 (16)
C9	0.069 (3)	0.064 (3)	0.044 (2)	−0.001 (2)	0.014 (2)	−0.009 (2)
C10	0.062 (3)	0.067 (3)	0.045 (2)	−0.011 (2)	0.005 (2)	−0.011 (2)
C11	0.053 (3)	0.067 (3)	0.044 (2)	−0.002 (2)	0.003 (2)	0.001 (2)
C12	0.056 (3)	0.053 (2)	0.042 (2)	0.001 (2)	0.014 (2)	−0.0004 (18)
C13	0.047 (2)	0.046 (2)	0.038 (2)	0.0000 (18)	0.0057 (18)	0.0031 (17)
C14	0.055 (3)	0.109 (4)	0.073 (3)	0.002 (3)	0.001 (3)	−0.010 (3)
C15	0.060 (3)	0.045 (2)	0.047 (2)	−0.001 (2)	0.009 (2)	0.0006 (19)
C16	0.067 (3)	0.052 (3)	0.052 (3)	−0.005 (2)	0.013 (2)	0.002 (2)
C17	0.067 (3)	0.069 (3)	0.079 (4)	−0.009 (3)	0.002 (3)	0.008 (3)
C18	0.074 (4)	0.088 (4)	0.091 (4)	−0.011 (3)	0.006 (3)	−0.002 (3)
C19	0.084 (4)	0.080 (4)	0.093 (4)	−0.013 (3)	0.013 (3)	0.010 (3)
C20	0.077 (4)	0.062 (3)	0.077 (3)	−0.004 (3)	0.023 (3)	0.021 (2)
C21	0.068 (3)	0.058 (3)	0.051 (2)	−0.002 (2)	0.011 (2)	−0.001 (2)
C22	0.067 (3)	0.048 (3)	0.051 (2)	0.005 (2)	0.012 (2)	0.0020 (19)
C23	0.080 (4)	0.064 (3)	0.077 (3)	0.004 (3)	0.025 (3)	0.025 (3)
C24	0.076 (4)	0.062 (3)	0.077 (3)	0.021 (3)	0.020 (3)	0.029 (3)
C25	0.077 (3)	0.062 (3)	0.054 (3)	0.012 (3)	0.008 (2)	−0.004 (2)
C26	0.064 (3)	0.049 (2)	0.059 (3)	0.003 (2)	0.012 (2)	−0.001 (2)
C27	0.062 (3)	0.049 (2)	0.045 (2)	0.003 (2)	0.010 (2)	0.0021 (19)
C28	0.074 (4)	0.101 (4)	0.087 (4)	0.029 (3)	−0.004 (3)	−0.003 (3)

### Tetrakis[8-methylphenanthridin-6(5H)-one-κO]bis(nitrato-κO)copper(II) Geometric parameters (Å, º)

**Table:**

Cu1—O2^i^	1.942 (2)	C10—H10A	0.9300
Cu1—O2	1.942 (2)	C11—C12	1.369 (6)
Cu1—O1	1.953 (2)	C11—C14	1.502 (6)
Cu1—O5	2.520 (3)	C12—C13	1.396 (5)
Cu1—O1^i^	1.953 (2)	C12—H12A	0.9300
O1—C1	1.264 (4)	C14—H14A	0.9600
O2—C15	1.245 (5)	C14—H14B	0.9600
O3—N3	1.212 (5)	C14—H14C	0.9600
O4—N3	1.237 (5)	C15—C27	1.446 (6)
O5—N3	1.279 (5)	C16—C17	1.390 (6)
N1—C1	1.331 (5)	C16—C21	1.400 (6)
N1—C2	1.403 (5)	C17—C18	1.391 (7)
N1—H1N	0.9000	C17—H17A	0.9300
N2—C15	1.339 (5)	C18—C19	1.393 (7)
N2—C16	1.401 (5)	C18—H18A	0.9300
N2—H2N	0.9001	C19—C20	1.392 (8)
C1—C13	1.460 (5)	C19—H19A	0.9300
C2—C3	1.389 (6)	C20—C21	1.388 (6)
C2—C7	1.394 (6)	C20—H20A	0.9300
C3—C4	1.392 (6)	C21—C22	1.437 (6)
C3—H3A	0.9300	C22—C23	1.385 (6)
C4—C5	1.377 (7)	C22—C27	1.420 (6)
C4—H4A	0.9300	C23—C24	1.409 (7)
C5—C6	1.371 (6)	C23—H23A	0.9300
C5—H5A	0.9300	C24—C25	1.364 (7)
C6—C7	1.401 (6)	C24—H24A	0.9300
C6—H6A	0.9300	C25—C26	1.398 (6)
C7—C8	1.444 (5)	C25—C28	1.490 (7)
C8—C9	1.401 (5)	C26—C27	1.385 (6)
C8—C13	1.403 (5)	C26—H26A	0.9300
C9—C10	1.362 (6)	C28—H28A	0.9600
C9—H9A	0.9300	C28—H28B	0.9600
C10—C11	1.399 (6)	C28—H28C	0.9600
			
O5···N2	2.806 (4)	C9···C15^ii^	3.197 (5)
O5···N1	2.852 (4)	C9···C27^ii^	3.308 (6)
H9A···O1^ii^	2.60	C6···H9A	2.71
O1···H12A	2.48	C7···H23A^iv^	2.90
O2···H26A	2.48	C9···H6A	2.72
O3···H12A^i^	2.58	C20···H23A	2.68
O4···H3A	2.68	C23···H20A	2.67
O4···H1N	2.64	H1N···H3A	2.40
O4···H26A^i^	2.72	H2N···H17A	2.39
O4···H4A^iii^	2.60	H6A···H9A	2.17
O5···H1N	1.97	H12A···H14A	2.37
O5···H2N	2.07	H14A···H14A^v^	2.39
N3···H1N	2.71	H20A···H23A	2.12
C6···C26^ii^	3.388 (7)	H24A···H28A	2.32
			
O2^i^—Cu1—O2	180.0	C12—C13—C8	120.8 (4)
O2^i^—Cu1—O1	90.74 (11)	C12—C13—C1	119.1 (4)
O2—Cu1—O1	89.26 (11)	C8—C13—C1	120.0 (4)
O2^i^—Cu1—O1^i^	89.26 (11)	C11—C14—H14A	109.5
O2—Cu1—O1^i^	90.74 (11)	C11—C14—H14B	109.5
O1—Cu1—O1^i^	180.00 (5)	H14A—C14—H14B	109.5
O1—Cu1—O5	90.79 (11)	C11—C14—H14C	109.5
O2—Cu1—O5	94.21 (11)	H14A—C14—H14C	109.5
C1—O1—Cu1	131.4 (2)	H14B—C14—H14C	109.5
C15—O2—Cu1	133.6 (3)	O2—C15—N2	120.7 (4)
C1—N1—C2	124.9 (4)	O2—C15—C27	121.4 (4)
C1—N1—H1N	115.1	N2—C15—C27	117.9 (4)
C2—N1—H1N	119.8	C17—C16—C21	122.8 (4)
C15—N2—C16	124.8 (4)	C17—C16—N2	118.7 (4)
C15—N2—H2N	115.1	C21—C16—N2	118.5 (4)
C16—N2—H2N	119.8	C16—C17—C18	119.2 (5)
O3—N3—O4	123.5 (4)	C16—C17—H17A	120.4
O3—N3—O5	119.9 (4)	C18—C17—H17A	120.4
O4—N3—O5	116.6 (4)	C17—C18—C19	119.3 (6)
O1—C1—N1	121.6 (4)	C17—C18—H18A	120.4
O1—C1—C13	121.1 (4)	C19—C18—H18A	120.4
N1—C1—C13	117.3 (3)	C20—C19—C18	120.3 (5)
C3—C2—C7	121.5 (4)	C20—C19—H19A	119.9
C3—C2—N1	119.2 (4)	C18—C19—H19A	119.9
C7—C2—N1	119.2 (4)	C21—C20—C19	121.9 (5)
C2—C3—C4	119.3 (4)	C21—C20—H20A	119.1
C2—C3—H3A	120.4	C19—C20—H20A	119.1
C4—C3—H3A	120.4	C20—C21—C16	116.6 (5)
C5—C4—C3	120.1 (5)	C20—C21—C22	123.9 (4)
C5—C4—H4A	120.0	C16—C21—C22	119.5 (4)
C3—C4—H4A	120.0	C23—C22—C27	117.6 (4)
C6—C5—C4	120.1 (4)	C23—C22—C21	123.0 (4)
C6—C5—H5A	120.0	C27—C22—C21	119.4 (4)
C4—C5—H5A	120.0	C22—C23—C24	120.2 (5)
C5—C6—C7	121.8 (4)	C22—C23—H23A	119.9
C5—C6—H6A	119.1	C24—C23—H23A	119.9
C7—C6—H6A	119.1	C25—C24—C23	122.0 (4)
C2—C7—C6	117.2 (4)	C25—C24—H24A	119.0
C2—C7—C8	118.7 (4)	C23—C24—H24A	119.0
C6—C7—C8	124.1 (4)	C24—C25—C26	118.4 (5)
C9—C8—C13	116.5 (4)	C24—C25—C28	119.9 (5)
C9—C8—C7	123.7 (4)	C26—C25—C28	121.7 (5)
C13—C8—C7	119.8 (4)	C27—C26—C25	120.7 (4)
C10—C9—C8	121.8 (4)	C27—C26—H26A	119.7
C10—C9—H9A	119.1	C25—C26—H26A	119.7
C8—C9—H9A	119.1	C26—C27—C22	121.0 (4)
C9—C10—C11	121.7 (4)	C26—C27—C15	119.3 (4)
C9—C10—H10A	119.1	C22—C27—C15	119.6 (4)
C11—C10—H10A	119.1	C25—C28—H28A	109.5
C12—C11—C10	117.4 (4)	C25—C28—H28B	109.5
C12—C11—C14	121.7 (4)	H28A—C28—H28B	109.5
C10—C11—C14	120.9 (4)	C25—C28—H28C	109.5
C11—C12—C13	121.7 (4)	H28A—C28—H28C	109.5
C11—C12—H12A	119.1	H28B—C28—H28C	109.5
C13—C12—H12A	119.1		
			
Cu1—O1—C1—N1	−29.9 (5)	Cu1—O2—C15—N2	−7.3 (6)
Cu1—O1—C1—C13	150.4 (3)	Cu1—O2—C15—C27	171.2 (3)
C2—N1—C1—O1	−178.8 (3)	C16—N2—C15—O2	171.4 (4)
C2—N1—C1—C13	0.9 (5)	C16—N2—C15—C27	−7.1 (6)
C1—N1—C2—C3	179.3 (4)	C15—N2—C16—C17	−175.2 (4)
C1—N1—C2—C7	−1.6 (6)	C15—N2—C16—C21	4.6 (6)
C7—C2—C3—C4	0.6 (6)	C21—C16—C17—C18	−0.7 (8)
N1—C2—C3—C4	179.7 (4)	N2—C16—C17—C18	179.1 (5)
C2—C3—C4—C5	−1.0 (7)	C16—C17—C18—C19	0.7 (8)
C3—C4—C5—C6	1.1 (7)	C17—C18—C19—C20	−0.6 (9)
C4—C5—C6—C7	−0.7 (7)	C18—C19—C20—C21	0.6 (9)
C3—C2—C7—C6	−0.2 (6)	C19—C20—C21—C16	−0.6 (7)
N1—C2—C7—C6	−179.3 (3)	C19—C20—C21—C22	−179.8 (5)
C3—C2—C7—C8	179.3 (4)	C17—C16—C21—C20	0.7 (7)
N1—C2—C7—C8	0.2 (5)	N2—C16—C21—C20	−179.1 (4)
C5—C6—C7—C2	0.3 (6)	C17—C16—C21—C22	179.9 (4)
C5—C6—C7—C8	−179.2 (4)	N2—C16—C21—C22	0.1 (6)
C2—C7—C8—C9	−175.5 (4)	C20—C21—C22—C23	−1.9 (7)
C6—C7—C8—C9	4.0 (6)	C16—C21—C22—C23	178.9 (4)
C2—C7—C8—C13	1.6 (5)	C20—C21—C22—C27	177.5 (4)
C6—C7—C8—C13	−178.9 (4)	C16—C21—C22—C27	−1.7 (6)
C13—C8—C9—C10	3.4 (6)	C27—C22—C23—C24	−2.0 (7)
C7—C8—C9—C10	−179.4 (4)	C21—C22—C23—C24	177.4 (5)
C8—C9—C10—C11	−1.7 (7)	C22—C23—C24—C25	1.0 (8)
C9—C10—C11—C12	−1.0 (6)	C23—C24—C25—C26	1.1 (8)
C9—C10—C11—C14	179.1 (5)	C23—C24—C25—C28	−179.0 (5)
C10—C11—C12—C13	1.9 (6)	C24—C25—C26—C27	−2.0 (7)
C14—C11—C12—C13	−178.3 (4)	C28—C25—C26—C27	178.1 (4)
C11—C12—C13—C8	−0.1 (6)	C25—C26—C27—C22	0.9 (6)
C11—C12—C13—C1	−177.7 (4)	C25—C26—C27—C15	−176.6 (4)
C9—C8—C13—C12	−2.5 (5)	C23—C22—C27—C26	1.1 (6)
C7—C8—C13—C12	−179.8 (4)	C21—C22—C27—C26	−178.4 (4)
C9—C8—C13—C1	175.1 (4)	C23—C22—C27—C15	178.6 (4)
C7—C8—C13—C1	−2.2 (5)	C21—C22—C27—C15	−0.9 (6)
O1—C1—C13—C12	−1.7 (5)	O2—C15—C27—C26	4.1 (6)
N1—C1—C13—C12	178.6 (3)	N2—C15—C27—C26	−177.4 (4)
O1—C1—C13—C8	−179.3 (3)	O2—C15—C27—C22	−173.5 (4)
N1—C1—C13—C8	1.0 (5)	N2—C15—C27—C22	5.1 (6)

Symmetry codes: (i) −*x*+1/2, −*y*+1/2, −*z*+1; (ii) −*x*+1/2, *y*−1/2, −*z*+1/2; (iii) −*x*+1, −*y*, −*z*+1; (iv) *x*, *y*−1, *z*; (v) −*x*, *y*, −*z*+1/2.

### Tetrakis[8-methylphenanthridin-6(5H)-one-κO]bis(nitrato-κO)copper(II) Hydrogen-bond geometry (Å, º)

*Cg*1, *Cg*3, *Cg*4 and *Cg*5 are the centroids of the (N1/C1/C2/C7/C8/C13), (C2–C7), (C8–C13) and (C16–C21) rings, respectively.

**Table:**

*D*—H···*A*	*D*—H	H···*A*	*D*···*A*	*D*—H···*A*
N1—H1*N*···O5	0.90	1.97	2.852 (4)	165
N2—H2*N*···O5	0.90	2.07	2.806 (4)	139
C4—H4*A*···O4^iii^	0.93	2.60	3.433 (6)	150
C9—H9*A*···O1^ii^	0.93	2.60	3.514 (5)	169
C12—H12*A*···O3^i^	0.93	2.57	3.455 (6)	158
C14—H14*C*···*Cg*5^vi^	0.96	2.92	3.768 (6)	147
C20—H20*A*···*Cg*3^vii^	0.93	2.86	3.644 (5)	143
C23—H23*A*···*Cg*1^vii^	0.93	2.89	3.726 (5)	151
C24—H24*A*···*Cg*4^vii^	0.93	2.77	3.557 (5)	143
C28—H28*C*···*Cg*3^viii^	0.96	2.88	3.716 (6)	146

Symmetry codes: (i) −*x*+1/2, −*y*+1/2, −*z*+1; (ii) −*x*+1/2, *y*−1/2, −*z*+1/2; (iii) −*x*+1, −*y*, −*z*+1; (vi) *x*, −*y*−1, *z*−1/2; (vii) *x*, *y*+1, *z*; (viii) *x*, −*y*, *z*−1/2.
